# Impact of training on waste management practices among healthcare workers

**DOI:** 10.6026/973206300213766

**Published:** 2025-10-31

**Authors:** Mayank Das, Sanchit Pradhan, Jyoti Adwani, Avnica Agarwal, Arunendra Singh Chauhan, Omveer Singh

**Affiliations:** 1 Department of Public Health Dentistry, Subharti Dental College and Hospital, Swami Vivekanand Subharti University, Meerut, Uttar Pradesh, India; 2 Department of Endocrine Surgery, King George's Medical University, Lucknow; 3 Department of Public Health Dentistry, ITS Dental College & Research Center, Greater Noida, Uttar Pradesh, India; 4 Department of Public Health Dentistry, Maharishi Markandeshwar College of Dental Sciences and Research, Mullana-Ambala, Haryana, India; 5 Department of Public Health Dentistry, Career Post Graduate Institute of Dental Sciences and Hospital, Lucknow, India

**Keywords:** Bio-medical waste, health care workers, infection control, quasi-experimental study, waste management practices, effectiveness, training

## Abstract

mproper biomedical waste management poses a major risk to infection control and public health. This quasi-experimental study among 100 healthcare workers from two institutes in Meerut assessed the effectiveness of training on waste management and infection control. Group A received structured lectures, while Group B received educational materials, with assessments at 1, 3 and 6 months. Significant improvements were observed in knowledge, attitude and practice scores over time (p<0.05). The training program effectively enhanced awareness and practices of healthcare workers toward infection control and waste management.

## Background:

Biomedical waste management (BWM) has emerged as a major global public health concern requiring urgent intervention. The term "BWM" is defined as any waste generated during diagnosis, treatment, or immunization of humans or animals, or during research and production/testing of biologicals, as per the Biomedical Waste (Management and Handling) Rules, 1998 by the Government of India [1]. Health care waste is broadly categorized into hazardous and non-hazardous types. Hazardous waste includes infectious materials, sharps, chemicals, pharmaceuticals and radioactive substances; whereas non-hazardous waste comprises items like paper and packaging that pose minimal risk. The World Health Organization reports that 85% of hospital waste is harmless, while 10% is infectious and 5% noninfectious but hazardous [2]. Studies indicate that 15-35% of hospital waste is infectious and demands strict regulation. Although developed nations have improved biomedical waste management (BMW) practices, developing countries continue to face significant gaps, resulting in increased transmission of hepatitis B, hepatitis C and other infections among patients and healthcare workers [3]. In India, hospitals generate approximately 1.3-1.45 kg of waste per patient per day-significantly lower than developed countries (up to 4.5 kg)-but improper segregation and disposal practices make management more challenging [4]. Poor handling of BMW poses health risks to healthcare workers, patients, waste handlers and even the surrounding community [5]. Therefore, effective BMW is a key component of hospital hygiene and infection control. Continuous training and awareness of BMW regulations among healthcare professionals are essential for maintaining safe and sustainable hospital environments [6]. Therefore, it is of interest to evaluate the effectiveness of training sessions in improving knowledge and practices related to biomedical waste management among healthcare professionals.

## Materials and Methods:

The quasi-experimental study was carried out in two health care institutes in Meerut city. Ethical clearance was obtained from the institutional ethical committee. Prior to the study, permission was taken from head of institutes. The study procedure was explained to study the subject and informed consent was taken from all participant of the study and confidentiality of the participants was maintained. The study was conducted in two health care institutes between February 2023 and July 2023. All the volunteers were invited for study after explanation of the study procedure and those who accept to participate in the study were included as per inclusion and exclusion criteria including 100 participants of both the institutes. The Exclusion criteria were participants who have already attended the training session and lecture on waste management and infection control and participants not willing to participate in the study. Simple random technique was used and sample size estimation was done by using G Power software (version 3.0). A minimum total sample size of 188 was found to be sufficient for an alpha of 0.05, power of 80% and 0.20 as effect size. Anticipating a dropout rate of 5% in this follow-up study, the total final sample size was decided as 200 (100 per group). A pilot study was conducted on 20 participants to check the validity of the questionnaire and the feasibility of the study. These subjects were not included in the final study. Inter-examiner reliability was calculated which was found to be fair as 0.70. The questionnaire was distributed to participants before and after the intervention process related to infection control and waste management. The components were self-assessment questions, knowledge-based and practice base questions related to infection control and waste management. The intervention consisted of a training sessions including lectures related to infection control and waste management. Educational health material related to biomedical waste management and infection control was distributed among participants of institutes. Among Group A participants, at the baseline knowledge attitude and practice questionnaire related to waste management and infection control. Reinforcement in form of lectures was given at 1 month, 3month. Follow up and assessment was done with the questionnaire at 1 month, 3 months and at the end of 6 months. Among Group B participants, at the baseline health educational material related to biomedical waste management and infection control, was given and again assessment with the questionnaire was done at 1 month, 3 months and at the end of 6 months. Data were analyzed using SPSS software (version 21). Intergroup comparison of increment in mean knowledge, attitude and practices scores was done by independent t-test while the intragroup comparison of mean knowledge, attitude and practices scores from baseline to different follow up points was done.

## Results:

The present study was conducted among 200 study participants among the experimental group and control group (100 in each group). In Table 1 (see PDF), the gender-wise distribution of study population among two study groups showed that both the study groups had an equal number of males and females and there were no gender wise differences. In Table 2 (see PDF), intragroup comparison of knowledge scores in the experimental group showed that there was a statistically significant increase in knowledge scores from 1 month to 3 months and from 3 months to 6 months. In Table 3 (see PDF), the intragroup comparison of attitude scores in the experimental group showed that there was a statistically significant increase in attitude scores from 1 month to 3 months and from 3 months to 6 months. Also, the intragroup comparison of attitude scores in the control group showed that attitude scores did not increase significantly from 1 month to 3 months, but it increased significantly from 3 months to 6 months. In Table 4 (see PDF), intragroup comparison of practices scores in the experimental group showed that there was a statistically significant increase in practices scores from 1 month to 3 months and from 3 months to 6 months. Also, intragroup comparison of practices scores in control group showed that practices scores did not show any significant increase from baseline to 1 month and from 1 month to 3 months and then it further increased significantly from 3 months to 6 months. However, intergroup comparison of practices scores between experimental and control group showed that at baseline, there was no statistically significant difference in the practices scores but at every follow up, at every point of time i.e. 1 month, 3 months and 6 months, the practices scores among experimental group was significantly higher than that among control group subjects.

## Discussion:

The findings from previous studies consistently highlight the critical importance of continuous education and structured training in improving biomedical waste management (BMWM) knowledge, attitude and practices among healthcare personnel. Studies have demonstrated that compliance with waste handling and disposal protocols remains suboptimal in many healthcare settings, particularly in waste transportation practices, which showed the lowest adherence rates [7]. However, the implementation of targeted training programs has been shown to significantly enhance post-test knowledge scores, reaffirming the effectiveness of educational interventions in improving compliance and awareness levels [7]. In the present study, intragroup comparison within the experimental group revealed a statistically significant increase in knowledge scores over successive time intervals, suggesting that sustained training and periodic reinforcement contribute to better knowledge retention and practical application. This aligns with previous evidence indicating that a large proportion of nursing and paramedical staff possess only moderate knowledge and display an unfavorable attitude toward BMWM before formal training [8]. Structured training sessions, therefore, serve as a pivotal component in transforming both cognitive understanding and behavioral attitudes [9]. Furthermore, research has shown that awareness of waste segregation protocols, especially color-coded categorization, remains inconsistent among healthcare workers, with less than three-quarters demonstrating adequate understanding or having undergone formal training [10]. Additional investigations in dental and hospital environments have also found significant associations between knowledge levels and specific factors such as familiarity with waste categories, disposal methods and regulatory oversight mechanisms [11]. These correlations underscore the need for standardized training across all cadres of healthcare professionals. Recent evidence from regional and global studies indicates that inadequate knowledge and poor waste-handling practices persist, especially among sanitary and auxiliary workers [12]. A lack of access to functional incineration facilities and limited staff training further exacerbate improper waste disposal, posing substantial risks to environmental and public health [13]. Generally, educational training plays a crucial role in improving healthcare workers' practices and compliance in healthcare waste management, leading to safer and more efficient handling of medical waste, though continued efforts are needed to sustain and optimize these improvements [14]. Educational interventions are effective in improving healthcare workers' knowledge and practices related to waste management, particularly when delivered as multi-component programs [15]. Collectively, these findings emphasize the necessity of integrating comprehensive BMWM and infection control modules into the curricula of medical, dental, nursing and allied health institutions. Continuous professional development, supported by regular assessments and refresher programs, is vital to maintaining adherence to regulatory standards and minimizing occupational and ecological hazards. Future research should adopt mixed-method and longitudinal approaches to evaluate the sustained impact of educational interventions on BMWM compliance and institutional safety culture [7, 8, 9, 10, 11, 12-13].

## Conclusion:

The training programme was found to be effective in improving the knowledge, attitude and practices regarding infection control and waste management among health care workers.

## Source of Funding:

NIL
